# A Live-Attenuated Equine Influenza Vaccine Stimulates Innate Immunity in Equine Respiratory Epithelial Cell Cultures That Could Provide Protection From Equine Herpesvirus 1

**DOI:** 10.3389/fvets.2021.674850

**Published:** 2021-06-10

**Authors:** Lila M. Zarski, Wendy E. Vaala, D. Craig Barnett, Fairfield T. Bain, Gisela Soboll Hussey

**Affiliations:** ^1^Department of Pathobiology and Diagnostic Investigation, College of Veterinary Medicine, Veterinary Medical Center, East Lansing, MI, United States; ^2^Merck & Co., Inc., Kenilworth, NJ, United States

**Keywords:** horse, EHV-1, equine influenza vaccine, epithelial cell, mucosal immunity

## Abstract

Equine herpesvirus 1 (EHV-1) ubiquitously infects horses worldwide and causes respiratory disease, abortion, and equine herpesvirus myeloencephalopathy. Protection against EHV-1 disease is elusive due to establishment of latency and immune-modulatory features of the virus. These include the modulation of interferons, cytokines, chemokines, antigen presentation, and cellular immunity. Because the modulation of immunity likely occurs at the site of first infection—the respiratory epithelium, we hypothesized that the mucosal influenza vaccine Flu Avert^®^ I.N. (Flu Avert), which is known to stimulate strong antiviral responses, will enhance antiviral innate immunity, and that these responses would also provide protection from EHV-1 infection. To test our hypothesis, primary equine respiratory epithelial cells (ERECs) were treated with Flu Avert, and innate immunity was evaluated for 10 days following treatment. The timing of Flu Avert treatment was also evaluated for optimal effectiveness to reduce EHV-1 replication by modulating early immune responses to EHV-1. The induction of interferons, cytokine and chemokine mRNA expression, and protein secretion was evaluated by high-throughput qPCR and multiplex protein analysis. Intracellular and extracellular EHV-1 titers were determined by qPCR. Flu Avert treatment resulted in the modulation of IL-8, CCL2, and CXCL9 starting at days 5 and 6 post-treatment. Coinciding with the timing of optimal chemokine induction, our data also suggested the same timing for reduction of EHV-1 replication. In combination, our results suggest that Flu Avert may be effective at counteracting some of the immune-modulatory properties of EHV-1 at the airway epithelium and the peak for this response occurs 5–8 days post-Flu Avert treatment. Future *in vivo* studies are needed to investigate Flu Avert as a prophylactic in situations where EHV-1 exposure may occur.

## Introduction

Equine herpesvirus 1 (EHV-1) ubiquitously infects horses worldwide. It is responsible for causing respiratory disease, late term abortion in pregnant mares, or the crippling neurologic disease equine herpesvirus myeloencephalopathy (EHM). Foals become infected within the first weeks or months of life, and infection results in respiratory illness (1–4). Like many other herpesviruses, EHV-1 then establishes a life-long latent infection within the neurons or local lymphoid tissues (5–8). Following primary infection, virus neutralizing (VN) antibodies can be detected in the serum and nasal mucosa and are associated with protection against clinical respiratory disease and nasal shedding upon re-exposure to the virus (9–12). However, high serum antibody levels following vaccination or infection do not correlate with protection from viremia or subsequent secondary disease manifestations, such as abortion or EHM (4, 9, 13). In contrast, high levels of cytotoxic lymphocyte (CTL) precursor frequencies are thought to be crucial for protection from viremia, abortion, and EHM (9, 12, 13).

In recent years, the study of innate immunity to EHV-1 has also gained interest—particularly because it is known that innate immune events are critical for establishing and shaping pathogen-specific adaptive immunity, including cytotoxic T-cell responses (14–17). As the first site of viral contact, the respiratory epithelium provides a physical barrier against inhaled pathogens. Tight junctions and respiratory epithelial integrity have been shown to be an important aspect of innate immune protection against EHV-1 infection (18). Furthermore, at this site, EHV-1 has been shown to upregulate pattern recognition receptors, such as TLR3 and TLR9. Activation of these receptors signals downstream production of antiviral cytokines and chemokines (19–21). Moreover, epithelial cells respond to EHV-1 infection by secreting interferons, which act as direct antivirals to limit viral replication (20–24). *In vitro*, EHV-1 has been shown to target the induction of the type I interferon response (25), and there appear to be differences in sensitivity to interferons between neuropathic and abortigenic strains of EHV-1 (26). In addition, lower IFNα responses in nasal secretions of horses during early EHV-1 infection may increase the likelihood of developing EHM, indicating a role for early interferon production at the epithelium in disease protection (24). In addition to direct antiviral molecules secreted by the epithelium, clearance of herpesviruses at the primary replication site requires the recruitment of other immune cells. EHV-1 infection of equine respiratory epithelial cells (ERECs) induces the expression of chemokines, such as IL-8, CCL2, CCL5, CXCL9, and CXCL10, and modulates chemotaxis (20–22, 27, 28). Interestingly, neuropathic strains of EHV-1 have been shown to selectively stimulate CXCR3 ligands CXCL9 and CXCL10 production by the respiratory epithelium to facilitate attraction and infection of CD4, CD8, and monocytic CD172+ cells (27). This specific shaping of the recruitment of leukocytes is instrumental in the protection against herpesviruses (14, 16, 29).

Interestingly, immunity following EHV-1 infection or vaccination is often insufficient, and the main reason for this is the immune-modulatory properties of the virus. Evasive strategies employed by EHV-1 include its ability to establish intracellular infection quickly to avoid detection by VN antibody (30–32). The intracellular nature of EHV-1 also explains the finding that high levels of precursor CTLs are more likely to correlate with protection from viremia and disease than those of serum VN titers (9, 12, 13). However, EHV-1 is also known to interfere with antigen presentation *via* the downregulation of MHC-I, which hampers optimal CTL activation (20, 22, 33–35). Ultimately though, most immune modulating events are likely to occur at the respiratory mucosa during initial infection with EHV-1. For example, the EHV-1 protein pUL56 is known to interfere with interferon production and also induce the expression of the anti-inflammatory gene IL-10 in ERECs (22). Additionally, EHV-1 selectively interferes with the chemotaxis of leukocytes to the respiratory epithelium (36) and modulates transfer *via* infected lymphocytes to the vascular endothelium (32). Specifically, the EHV-1 protein pUL56 is known to modulate chemokine expression and neutrophil and monocyte chemotaxis in ERECs (22). Glycoprotein G (gG) of EHV-1 has chemokine binding properties, which has been shown to interfere with IL-8-mediated chemotaxis of neutrophils *in vitro* (37, 38). Furthermore, in a murine *in vivo* model of EHV-1 infection, gG has been shown to interfere with chemotaxis of inflammatory cells to the lung allowing for increased viral replication at this site (38, 39). Collectively, these immune evasive strategies of EHV-1, particularly during early infection, contribute to virulence *in vivo* and the inability of horses to develop lasting protective immunity following infection or vaccination.

Because early innate immunity is important for immediate protection as well as shaping adaptive immunity, counteracting immune modulation by EHV-1 during early epithelial infection could lead to a more robust innate immune response and consequently increased protective downstream adaptive immunity. To accomplish this, intranasal administration of live intranasal vaccines is an attractive method to stimulate mucosal innate immunity. These vaccines interact directly at the epithelium and are known to stimulate important features of mucosal innate immunity, including the secretion of inflammatory cytokines and chemokines, as well as the promotion of maturation of resident antigen presenting cells (40). For viral vaccines, attenuated viruses are often able to induce antiviral innate immune responses because they retain their ability to replicate at the epithelium and it is known that live viral replication in the respiratory tract in horses is a potent stimulator of mucosal immunity (41, 42).

Together with EHV-1, equine influenza virus (EIV) is a primary respiratory pathogen of horses. However, the fitness of influenza viruses relies to a large extent on their ability to change through antigenic drift and shift. EIV infection in horses is known to induce powerful mucosal immune responses (41–43). Furthermore, in humans, it has been shown that both live-attenuated influenza vaccine (LAIV) virus and wild-type influenza virus induce the upregulation of pattern recognition receptors, interferons, and chemokines in the nasal mucosa as well as in the respiratory epithelial cell culture systems, ultimately contributing to a diverse and potent adaptive immune response (14, 16, 41–49).

Furthermore, there is some clinical evidence that vaccination of weanling foals with an equine LAIV is associated with better respiratory health during weaning (50), and that this could be due to the LAIV inducing antiviral mucosal immunity. The LAIV used in this study was Flu Avert^®^ I.N. (Flu Avert) (MSD Animal Health, Kenilworth, NJ, USA), which is a commercially available equine LAIV, that has been proven to be safe and efficacious at protecting horses against EIV (51–53). We hypothesize that Flu Avert induces innate immune responses at the respiratory epithelium that could potentially overcome some of the immune-modulatory events that occur during EHV-1 infection and thus also provide protection against EHV-1 infection.

## Materials and Methods

### Experimental Design

For this study, three experiments were conducted. A preliminary experiment evaluated the toxicity of Flu Avert in ERECs over a period of 7 days. The second experiment investigated the effects of Flu Avert treatment on innate epithelial immune responses for 10 days after treatment of ERECs. A final experiment evaluated the effect of Flu Avert treatment for protection from EHV-1 and modulation of innate immune responses at varying times (1, 2, 5, or 7 days) prior to EHV-1 infection of ERECs. In this final experiment, EHV-1 viral titers as well as cytokine and chemokine mRNA and protein gene expression were evaluated.

### Viruses

Flu Avert was propagated from Flu Avert^®^ I.N.^A^ commercial stock inoculated into Madin-Darby Canine Kidney (MDCK) cells with media consisting of Eagle's Minimum Essential Medium (MEM, M5650; Sigma Aldrich, St. Louis, MO, USA) supplemented with 0.3% Bovine Serum Albumin (A3059; Sigma Aldrich, St. Louis, MO, USA), 1% GlutaMAX (GIBCO, Life Technologies, Carlsbad, CA, USA), 100 IU/ml penicillin, 100 μg/ml streptomycin, and 1 μg/ml trypsin. Cells were incubated at 33°C and 5% CO_2_ for 3–5 days until 90% of cells showed cytopathic effect (CPE). Cells and supernatants were collected, frozen to lyse the cells, thawed, and then centrifuged at 300 × *g* to remove cell debris, and the stock was stored at −80°C. The second passage was used for the experiments.

The Flu Avert titer was determined by plaque assay using serial dilutions of the propagated Flu Avert I.N. stock. Six-well plates seeded with MDCK cells were incubated with viral inoculum dilutions for 1 h at 33°C and 5% CO_2_ after which 1.5% methylcellulose media was added and plates were incubated for an additional 4–5 days until plaques developed. The cells were fixed and stained with a 2% crystal violet/6% formalin solution. Viral plaques were counted, and the titer was expressed as plaque forming unit (pfu) per ml of original strength virus stock.

The EHV-1 strain Ab4 (GenBank Accession No. AY665713.1) was propagated in rabbit kidney 13 (RK-13) cells with MEM-10 (Sigma Aldrich) supplemented with 100 IU/ml penicillin, 100 μg/ml streptomycin, 1% GlutaMAX (Gibco), and 10% fetal bovine serum (FBS) and incubated at 37°C and 5% CO_2_. After viral propagation, the cell culture supernatants were collected, clarified, and stored as described above. The EHV-1 titer was determined by plaque assay using 10-fold serial dilutions of virus stock in RK-13 cells incubated at 37°C and 5% CO_2_ using the method described above.

### Animals and EREC Cultures

Upper respiratory tract tissues were harvested from eight horses (mean age 15 years; range 6–23 years) that were euthanized *via* intravenous overdose of pentobarbital sodium (≥86 mg/kg) for reasons unrelated to respiratory disease. All procedures were performed in compliance with the Institutional Animal Care and Use Committee of Michigan State University. Primary ERECs were isolated and cryopreserved as previously described (21). Briefly, 6–8 inch sections of the trachea were dissected from horses immediately following euthanasia, and the mucosal surface was digested for 3–5 days at 4°C in a 1.4% Pronase (Roche Applied Science, Indianapolis, IN, USA) and 0.1% deoxyribonuclease I (Roche Applied Science) solution in MEM without calcium or magnesium (Sigma). Following digestion, the cells were incubated for 2 h in an uncoated Petri dish at 37°C to reduce fibroblast contamination after which the floating cells were collected and cryopreserved in liquid nitrogen until further use.

For each experiment, ERECs were thawed and cultured at the air–liquid interface by adding 2–3 million cells in 500 μl of DMEM/F12 (Gibco) media supplemented with 5% FBS that was not heat inactivated, 1% MEM non-essential amino acid solution (Gibco), 100 IU/ml penicillin, 100 μg/ml streptomycin, and 1.25 μg/ml amphotericin to the top chamber of a collagen-coated Transwell polyester membrane insert of a 12-well plate (Corning, Inc., Corning, NY, USA) as previously described (21). One ml of the above media was added to the bottom chamber, and the plates were incubated overnight at 37°C with 5% CO_2_. On the next day, the media in the top chamber was aspirated off, and the media in the bottom chamber was replaced with DMEM/F12^C^ supplemented with 2% Ultroser G TM (Pall, Port Washington, NY, USA), 100 IU/ml penicillin, 100 μg/ml streptomycin, and 1.25 μg/ml amphotericin B. The media in the bottom chamber was replaced every 2–4 days, and the cultures were maintained for 3–4 weeks until fully differentiated. Fully differentiated cultures were used in the subsequent experiments.

### Toxicity of Flu Avert in ERECs

#### Cell Culture and Inoculation

Fully differentiated EREC cultures derived from tracheal tissues from three horses were washed with 500 μl DMEM/F12 media (Gibco). Cells were treated with Flu Avert I.N. [with multiplicities of infection (MOIs) of 0.1, 1, or 5] or media control (MOI of 0) at the apical side of the cell culture, in 500 μl DMEM/F12 media (Gibco). After 2 h of incubation at 37°C with Flu Avert, the inoculum was removed, and cells were washed twice with DMEM media (Gibco) and incubated at 37°C until cell collection. Cell pellets were collected 1, 2, 3, 4, 5, 6, and 7 days post-Flu Avert treatment, and media supernatants in the bottom chamber were replaced every 4 days throughout the experiment. To collect the ERECs, cells were incubated with Accumax dissociation solution (Qiagen, Hilden, Germany) for 2 cycles. For this, the top chamber was rinsed with 300 μl phosphate-buffered saline (PBS), aspirated, and then incubated with 300 μl Accumax for 30 min at 37°C. The dissociation solution was then collected on ice, and another 300 μl Accumax was added and further incubated for 20–45 min until all remaining cells were dissociated and this solution was added to the first aliquot. The top chamber was rinsed again with 300 μl PBS to collect any remaining cells and added to the Accumax/cell suspension. The cells were then pelleted by spinning at 300 × *g* for 10 min.

#### Microscopic Evaluation

Cultures for the toxicity experiment were evaluated by microscopy for evidence of CPE daily for 7 days following Flu Avert treatment. Images were taken and scored using the scale described in Table 1.

**Table 1:** Grading scale for cytopathic effect.

**Grade**	**Criteria**
0	Cells appear healthy
1	Cell clumping or mucus in <50% of well or minor clumping/mucus
2	Cell clumping or mucus in >50% of well or severe clumping/mucus
3	Same as grade “2” plus cells peeling off of culture membrane

#### Cell Viability Analysis

For the Flu Avert toxicity experiments, cell pellets were resuspended in PBS with 0.4% bovine serum albumin and 0.1% sodium azide and analyzed immediately following collection for cell viability using propidium iodide (PI) staining and flow cytometry. Twenty thousand events were collected from each sample both prior to and after staining with PI (10 μg/ml). Positive events in stained and unstained samples were determined using frequency gating in Flowing Software version 2.5.1. The percent of dead cells for each sample was determined from the percent of positive events for the PI-stained samples.

### Effects of Flu Avert Treatment on Immune Response in ERECs

Fully differentiated ERECs isolated from tracheal tissues of five horses were treated with Flu Avert (MOI = 5) or media control on the apical side in 500 μl DMEM/F12 media (Gibco). After 2 h of incubation at 37°C with Flu Avert or media, the inoculum was removed, and cells were washed twice with DMEM media (Gibco). Cells were incubated at 37°C and 5% CO_2_, and the media was changed every 2–4 days until sample collection. ERECs were collected using Accumax dissociation solution (Qiagen) daily 2–10 days post-Flu Avert or media treatment, as described in the previous experiment. The cell culture supernatants were collected 4, 5, 8, and 10 days post-treatment for protein cytokine analysis. Cells were divided into half, and the pellets were stored as two separate aliquots at −80°C until further processing for cytokine mRNA expression analyses. At the same time, the cell culture supernatants were collected into separate aliquots and stored at −80°C until further cytokine protein expression analyses.

### Effects of Flu Avert Treatment on EHV-1 Inoculation in ERECs

Fully differentiated ERECs isolated from tracheal tissues of five horses were treated with Flu Avert (MOI = 5) or media control on the apical side of the cell culture as described above. After 2 h of incubation at 37°C with Flu Avert or media, the inoculum was removed, and cells were washed twice with DMEM media (Gibco). Cells were incubated at 37°C and 5% CO_2_, and the media was changed every 2–4 days until EHV-1 inoculation.

Following Flu Avert or media treatment, ERECs were inoculated with EHV-1 strain Ab4 (MOI = 1) suspended in 500 μl DMEM/F12 (Gibco) or media at 1, 2, 5, or 7 days post-Flu Avert treatment. This dose was chosen based on previous studies [(18, 26); Zarski et al. in preparation], and the dose of the same virus was typically used by our group in challenge infection experiments of horses (54, 55). After 2 h of incubation at 37°C with EHV-1 or media, the inoculum was removed, and cells were washed once with DMEM/F12 media (Gibco) and incubated at 37°C until collection. ERECs were collected using Accumax dissociation solution (Qiagen) at 24, 48, and 72 h post-EHV-1/Mock inoculation as described in the previous experiment. Cells were divided into half, and the pellets were stored as two separate aliquots at −80°C until further processing for EHV-1 growth curves or cytokine mRNA expression analyses. At the same time, the cell culture supernatants were collected into separate aliquots and stored at −80°C until further processing for EHV-1 growth curves and cytokine protein expression analyses.

### EHV-1 Intracellular and Extracellular Growth Curves

As part of the final experiment, EHV-1 viral load was determined from the cell pellets (intracellular) and supernatants (extracellular) by quantitative real-time polymerase chain reaction (qPCR) for the EHV-1 gB gene as previously described (56). DNA was isolated from all samples using the MagAttract 96 cador Pathogen Kit (Thermo Fisher, Waltham, MA, USA) and quantified using the NanoDrop 2000 spectrophotometer (Applied Biosystems, Waltham, MA, USA). Reactions consisted of 10 μl TaqMan™ Fast Universal PCR Master Mix (2×), no AmpErase™ UNG (Takara Bio Inc., Kusatsu, Shiga, Japan), 400 nM forward and reverse primers, and 200 nM probe, with nuclease-free water and DNA template added to a final reaction volume of 20 μl. Plasmid DNA gene copies generated from the gB gene product were quantified and included in 10-fold serial dilutions in each run, in order to create a standard curve. Samples and standards were run in triplicate and duplicate, respectively. No-template controls were included on each plate. Thermocycling was performed on the Applied Biosystems 7500 Fast Real-Time PCR system using the following conditions: 20 s holding stage at 95°C, followed by 37 cycles of 3 s at 95°C denaturation and 30 s at 60°C annealing/extension. Viral load was expressed as EHV-1 copy number per ng of DNA for cell pellets and EHV-1 copy number per PCR reaction for supernatants.

### Cytokine Protein Expression in EREC Supernatants

Cytokine protein expression for equine cytokines IL-4, IL-10, IL-17, IFNα, and IFNγ in cell culture supernatants was evaluated using a bead-based multiplex assay as previously described by Wagner and Freer (57) at the Animal Health Diagnostic Center, Cornell University, Ithaca, NY, USA.

### mRNA Isolation

For gene expression analysis, cell pellets were lysed and homogenized using TRIzol reagent (Applied Biosystems) following the manufacturer's instructions. The aqueous phase was then collected and washed with 100% ethanol, and the RNA was isolated using the RNeasy Mini Kit (Thermo Fisher) according to the manufacturer's instructions. To eliminate genomic DNA contamination, deoxyribonuclease treatment (Thermo Fisher) was applied to each sample according to the manufacturer's recommendation.

### Reverse Transcription-Real Time Quantitative Polymerase Chain Reaction (RT-qPCR) for mRNA Expression Analysis

RNA was quantified using the NanoDrop 2000 spectrophotometer (Applied Biosystems), and 297 ng RNA was used in a 30 μl reverse transcription reaction with the High-Capacity cDNA Reverse Transcription Kit with RNAse inhibitor (Applied Biosystems). High-throughput qPCR was then performed using the SmartChip Real-Time PCR System (Takara Bio Inc.). The chip was loaded, and thermocycling was performed following the manufacturer's recommendations—with reactions consisting of template cDNA, TaqMan Gene Expression Master Mix (Applied Biosystems), and the appropriate primer/probe combination. See Table 2 for details on primers and probes. Samples were run in triplicate, with 12 no-template control reactions per chip. The raw mRNA expression data were examined for outliers. If sample triplicates had a variability >1.5 ct from each other, data points were excluded as the variability in technical replicates was considered to be too high to be accurate.

**Table 2:** Primer and probe source list.

**Gene**	**Source**
CCL2	TaqMan^®^ gene expression assay no: Ec03468496_ml (Thermo Fisher)
CCL5	TaqMan^®^ gene expression assay no: Ec03468106_ml (Thermo Fisher)
CXCL9	TaqMan^®^ gene expression assay no: Ec03469470_ml (Thermo Fisher)
CXCL10	TaqMan^®^ gene expression assay no: Ec03469403_ml (Thermo Fisher)
IL-8	TaqMan^®^ gene expression assay no: Ec03468860_ml (Thermo Fisher)
IFN-γ	TaqMan^®^ gene expression assay no: Ec03468606_ml (Thermo Fisher)
IFN-α	Designed in house; Forward 5′-CGGAAGCCTCAAGCCATCT-3′
	Reverse 5′-TCTGTGCTGAAGAGGTGGAAGA-3′
	Probe 5′-TGCGGTCCATGAGACGATCCAACA-3′
IFN-β	Young Go et al. (58)
IL-10	TaqMan^®^ gene expression assay no: Ec03468647_ml (Thermo Fisher)
ACTB	TaqMan^®^ gene expression assay no: Ec04176172_gH (Thermo Fisher)
B2M	TaqMan^®^ gene expression assay no: Ec03468699_ml (Thermo Fisher)
GUSB	TaqMan^®^ gene expression assay no: Ec03470630_m1 (Thermo Fisher)

Three housekeeping genes (ACTB, B2M, and GUSB) were used to normalize the genes of interest, and the average of the untreated and mock-inoculated cells was used as a calibrator. Relative expression was expressed as log fold change (the –ddCq value) from the calibrator for each gene of interest as described by Livak and Schmittgen (59).

### Statistical Analysis

All statistical analysis was performed using R software version 3.4.2. For cell viability analysis, differences in viability between the MOIs were analyzed using the Kruskal–Wallis rank sum test (kruskal.test function). For analyzing the effect of Flu Avert treatment on cytokine responses in ERECs, statistical analysis was performed using either a Wilcoxon rank sum test (wilcox.test function) for RT-qPCR data or a Welch's two sample *t*-test (t.test function) for protein data. For analyzing the effect on Flu Avert treatment and subsequent EHV-1 inoculation on cytokine responses in ERECs, statistical analysis was performed using either a Kruskal–Wallis rank sum test with Dunn's *post-hoc* analysis (dunn.test function, part of the dunn.test package) for RT-qPCR data or ANOVA (aov function) with Tukey's *post-hoc* analysis (TukeyHSD function) for protein expression. For EHV-1 replication, viral copy number in Flu Avert- and media-treated cells was compared using a Wilcoxon rank sum test (wilcox.test).

## Results

### Flu Avert Is Not Toxic in ERECs

**Figure 1:**
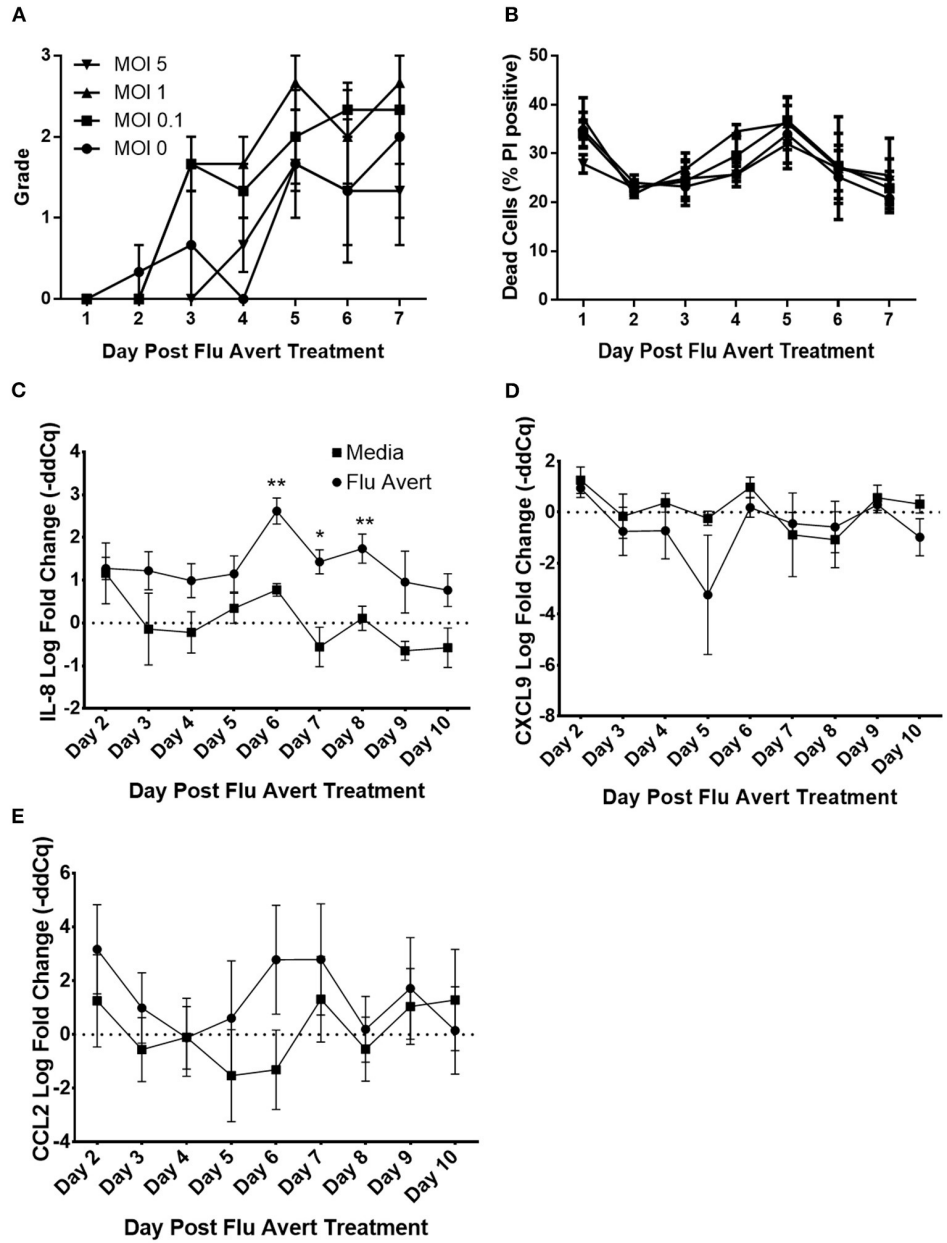
Cell viability and chemokine mRNA expression following Flu Avert treatment in ERECs. **(A)** Microscopic analysis of EREC cultures. Mean grades from cells ± SEM of three horses following Flu Avert treatment at different MOIs. Grading of cell viability from 0 to 3 is described in Table 1. **(B)** Cell viability analysis. Mean percent positive cells ± SEM of three horses at each MOI as determined with propidium iodide staining. Black circle is MOI = 0. Black square is MOI = 0.1. Black upright triangle is MOI = 1. Black upside-down triangle is MOI = 5. **(C)** IL-8 mRNA expression. **(D)** CXCL9 mRNAs expression. **(E)** CCL2 mRNA expression. Values are mean log fold change (–ddCq) ± SEM. Black square represents untreated (media) treated ERECs. Black circle represents Flu Avert-treated ERECs. *p ≤ 0.05 and **p < 0.01, respectively, between the media and Flu Avert treatment groups.

Before evaluation of the effect of Flu Avert on subsequent inoculation with EHV-1, we wanted to ensure that Flu Avert was not toxic for the EREC cultures over several days following Flu Avert inoculation. According to microscopic evaluation, there was no dose effect of Flu Avert on CPE scores (Figure 1A). A slight rise in score over time was observed for all doses including the media control (MOI = 0). PI viability staining confirmed the microscopic observations; there were no significant differences between groups, indicating that there was no toxic effect of Flu Avert when used up to an MOI of 5 for 7 days following treatment (Figure 1B).

### Flu Avert Induces Cytokine Responses in ERECs Between Days 5 and 10 Post-inoculation

Cytokine mRNA expression in Flu Avert-treated and untreated cells was evaluated to determine the effects of Flu Avert on stimulation of immune gene expression over time in ERECs. CCL2, CCL5, CXCL9, CXCL10, and IL-8 mRNA expression was detected in the samples. Interestingly, there were no statistically significant differences in cytokine expression between Flu Avert and media-treated cells until 6 days post-treatment. On days 6–8 post-treatment, IL-8 mRNA was significantly upregulated in Flu Avert-treated cells (Figure 1C). CXCL9 was downregulated in Flu Avert-treated cells when compared with media-treated cells on day 5 (*p* = 0.06) and day 10 (*p* = 0.11; Figure 1D). On day 6 post-treatment, CCL2 was upregulated, but this trend was not statistically significant (Figure 1E). There were no differences observed in CXCL10 and CCL5 expression (data not shown). Expression for IFNα, INFβ, IFNγ, and IL-10 mRNA was positive in <10% of the total samples (data not shown).

Protein secretion in EREC supernatants was also measured. IL-10 and IL-17 proteins were detected, but there were no differences between Flu Avert and media treatments (data not shown). Protein levels for IFNα and IL-4 were below the limit of detection for the assay, and levels of IFNγ were below 4 U/ml for all samples (data not shown).

### Reduction of EHV-1 Replication Corresponds With Timing of Peak Cytokine Responses in ERECs

**Figure 2:**
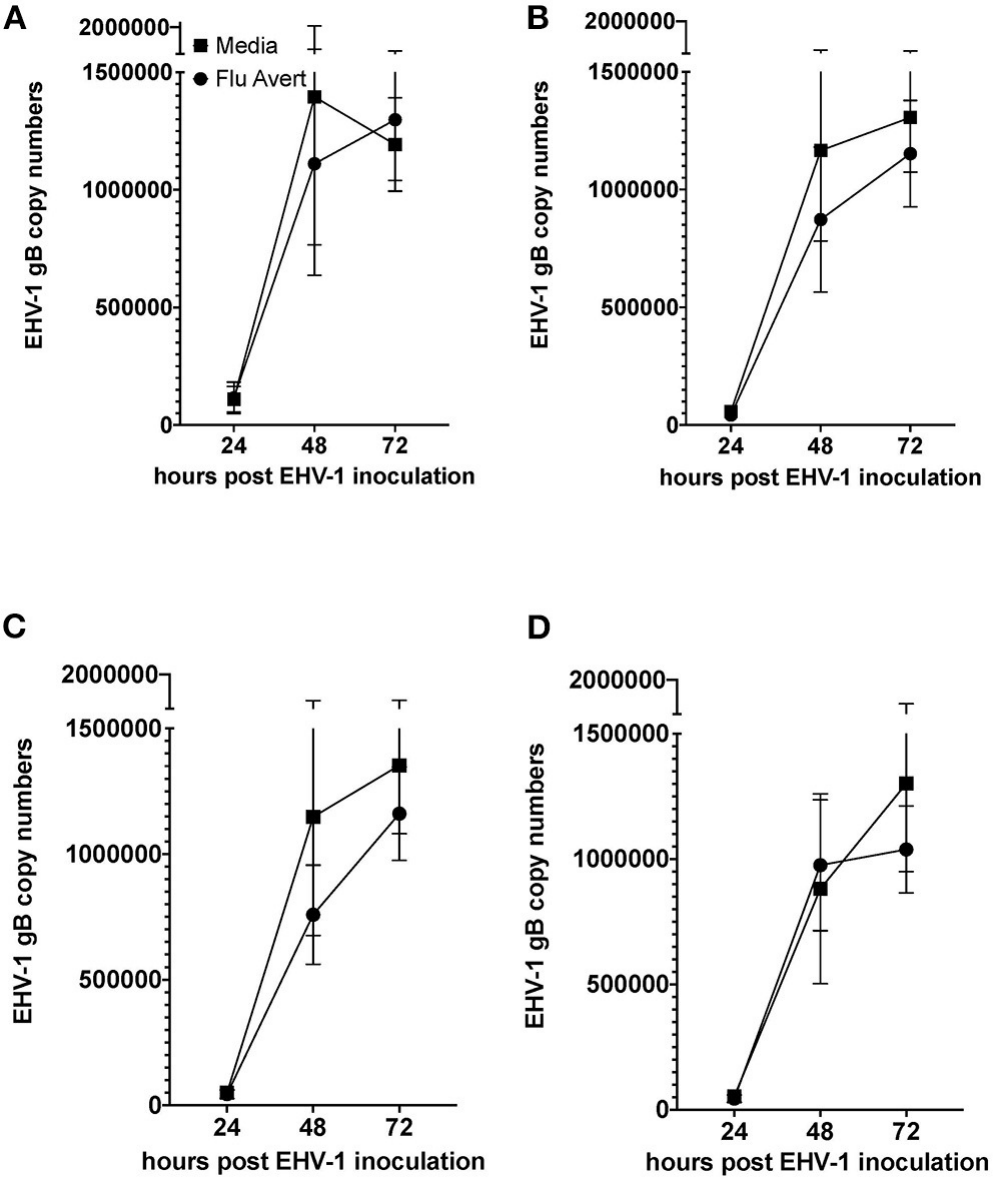
Mean ± SEM difference in intracellular EHV-1 copy number between Flu Avert-treated and media-treated ERECs from five horses. **(A)** Intracellular copy number in ERECs treated with Flu Avert or media on day 1 prior to EHV-1 inoculation. **(B)** Intracellular copy number in ERECs treated with Flu Avert or media on day 2 prior to EHV-1 inoculation. **(C)** Intracellular copy number in ERECs treated with Flu Avert or media on day 5 prior to EHV-1 inoculation. **(D)** Intracellular copy number in ERECs treated with Flu Avert or media on day 7 prior to EHV-1 inoculation.

**Figure 3:**
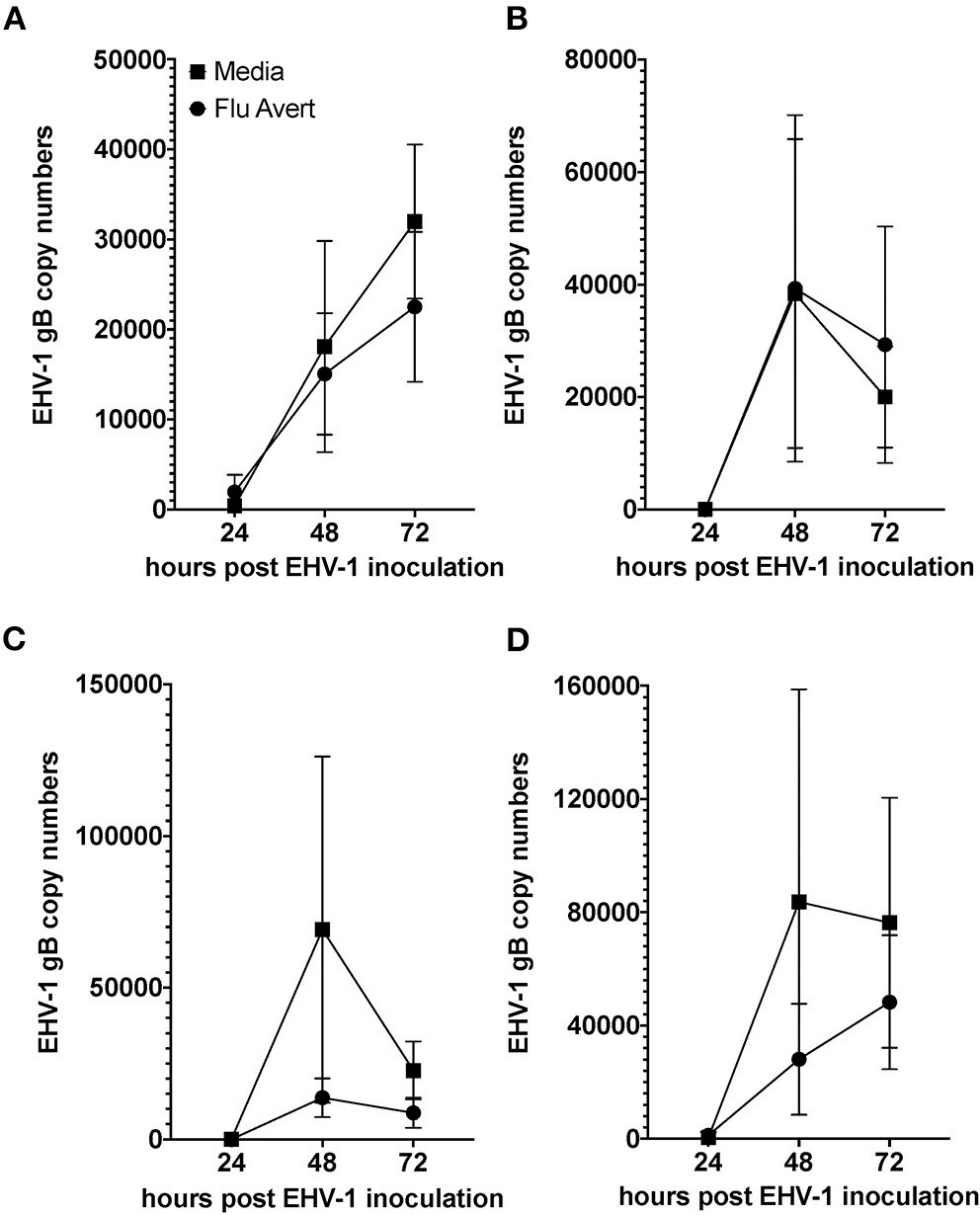
Mean ± SEM difference in extracellular EHV-1 copy number between Flu Avert-treated and media-treated ERECs from five horses. **(A)** Extracellular copy number in cells treated with Flu Avert or media on day 1 prior to EHV-1 inoculation. **(B)** Extracellular copy number in cells treated with Flu Avert or media on day 2 prior to EHV-1 inoculation. **(C)** Extracellular copy number in cells treated with Flu Avert or media on day 5 prior to EHV-1 inoculation. **(D)** Extracellular copy number in cells treated with Flu Avert or media on day 7 prior to EHV-1 inoculation.

EHV-1 viral load in Flu Avert-treated and untreated EHV-1-inoculated ERECs was determined for cell pellets (intracellular) and for supernatants (extracellular). In all EHV-1-inoculated cultures, few classical EHV-1 plaques were observed microscopically starting at 24 h post-infection, but plaques increased in number as time progressed (data not shown). By 48 h post-inoculation, EHV-1 plaques were observed in all wells microscopically, and significant EHV-1 titers were detected by qPCR in all EHV-1-inoculated wells irrespective of prior treatment with Flu Avert (Figures 2, 3). EHV-1 plaques and titers were also detectable at 72 h post-inoculation in all EHV-1-inoculated wells (Figures 2, 3). While no differences were observed in titers at 24 h post-inoculation, at 48 and 72 h post-EHV-1 inoculation, reduction in intracellular and extracellular titers was observed in cells treated with Flu Avert on individual days. However, these differences were not statistically significant. Because we wanted to further investigate if there was a timepoint of Flu Avert treatment that was most likely to consistently reduce EHV-1 replication across timepoints post-EHV-1 inoculation combined, culture wells in which EHV-1 titers were lower in Flu Avert-treated cultures than in untreated cultures post-EHV-1 inoculation were counted. Flu Avert treatment on day 5 resulted in the most cultures with reduced EHV-1 titers following Flu Avert treatment (8/10 wells for intracellular titers and 7/10 wells for extracellular titers) (Table 3). Taking the combined results in consideration, our data suggest that treatment with Flu Avert on day 5 prior to EHV-1 inoculation may be the most effective in reducing intracellular and extracellular EHV-1 titers in ERECs, although this data should be followed up with experiments in the natural host. All mock-inoculated samples were negative for EHV-1 (data not shown). Four mock-inoculated intracellular samples were excluded from analysis due to insufficient DNA isolation or contamination.

**Table 3:** Number of cultures in which EHV-1 viral load was reduced by Flu Avert treatment.

**Flu Avert treatment day**	**# of cultures where viral load is reduced by >10^**4**^ gB DNA copies**
**Intracellular virus**	
Day 1	7/10
Day 2	8/10
Day 5	8/10
Day 7	4/10
**Flu Avert treatment day**	**# of cultures where viral load is reduced by >500 gB DNA copies**
**Extracellular virus**	
Day 1	7/10
Day 2	5/10
Day 5	7/10
Day 7	6/10

### Flu Avert Treatment Enhances Cytokine Response to EHV-1 Infection in ERECs

**Figure 4:**
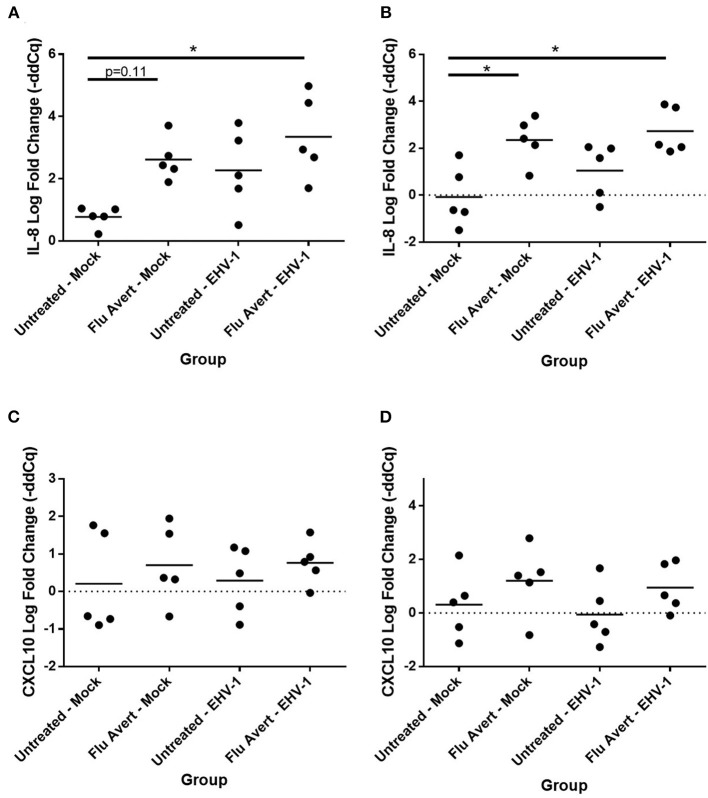
Effect of Flu Avert treatment on chemokine mRNA expression in ERECs collected from five horses 24 h following EHV-1 inoculation. **(A)** IL-8 mRNA expression in ERECs that were treated with Flu Avert on day 5 prior to EHV-1 inoculation. **(B)** IL-8 mRNA expression in ERECs that were treated with Flu Avert on day 7 prior to EHV-1 inoculation. **(C)** CXCL10 mRNA expression in ERECs that were treated with Flu Avert on day 5 prior to EHV-1 inoculation. **(D)** CXCL10 mRNA expression in ERECs that were treated with Flu Avert on day 7 prior to EHV-1 inoculation. The mean log fold change (–ddCq) is represented by a bar (*p < 0.05).

At 24 h post-EHV-1 inoculation in untreated cells, a statistically significant induction of chemokine responses was not observed. In contrast, when ERECs were pretreated with Flu Avert treatment on days 5 or 7 prior to EHV-1 inoculation, IL-8 expression was significantly upregulated in Flu Avert/EHV-1-inoculated ERECs, compared with the untreated and uninfected ERECs (Figures 4A,B). A similar trend was observed for CXCL10 expression in ERECs treated with Flu Avert 7 days prior to EHV-1 or mock inoculation, although this trend was not statistically significant (Figures 4C,D). There were no differences in IL-8 and CXCL10 expression for treatment days 1 or 2 (data not shown). No differences were observed between groups for expression of CCL2, CCL5, or CXCL9 at 24 h post-EHV-1 inoculation (data not shown).

**Figure 5:**
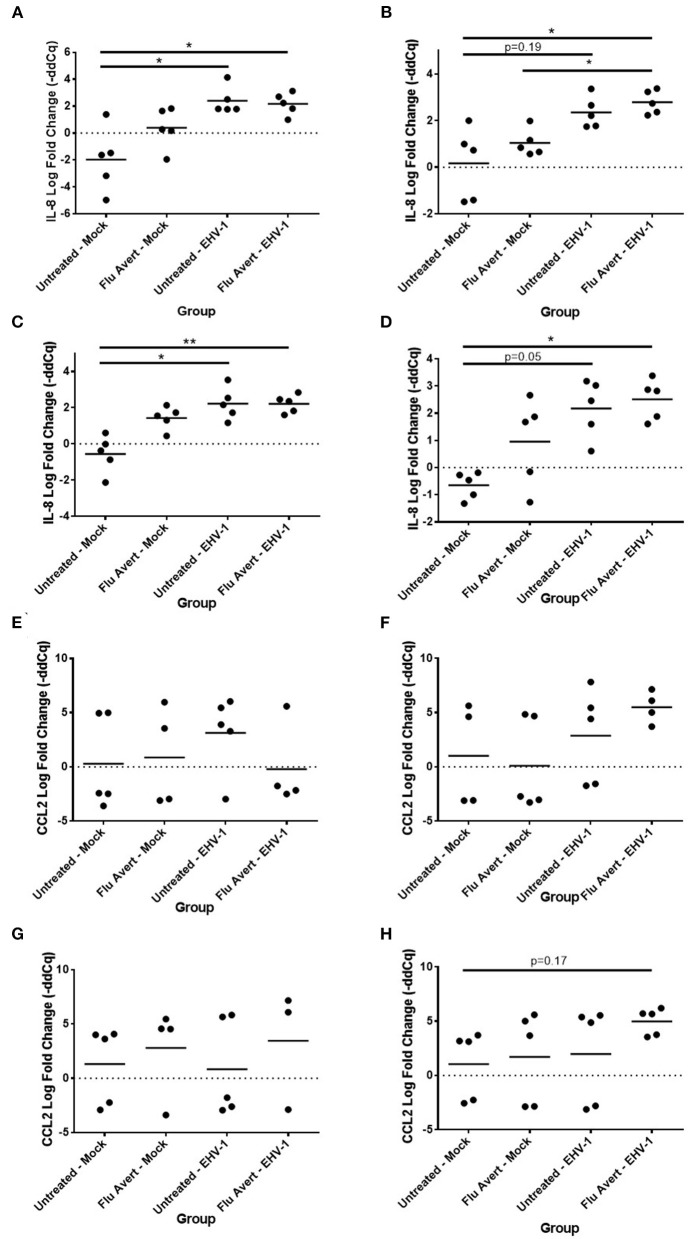
Effect of Flu Avert treatment on chemokine mRNA expression in ERECs collected from five horses 48 h following EHV-1 inoculation. **(A)** IL-8 mRNA expression in ERECs that were treated with Flu Avert on day 1 prior to EHV-1 inoculation. **(B)** IL-8 mRNA expression in ERECs that were treated with Flu Avert on day 2 prior to EHV-1 inoculation. **(C)** IL-8 mRNA expression in ERECs that were treated with Flu Avert on day 5 prior to EHV-1 inoculation. **(D)** IL-8 mRNA expression in ERECs that were treated with Flu Avert on day 7 prior to EHV-1 inoculation. **(E)** CCL2 mRNA expression in ERECs that were treated with Flu Avert on day 1 prior to EHV-1 inoculation. **(F)** CCL2 mRNA expression in ERECs that were treated with Flu Avert on day 2 prior to EHV-1 inoculation. **(G)** CCL2 mRNA expression in ERECs that were treated with Flu Avert on day 5 prior to EHV-1 inoculation. **(H)** CCL2 mRNA expression in ERECs that were treated with Flu Avert on day 7 prior to EHV-1 inoculation. The mean log fold change (–ddCq) is represented by a bar (**p < 0.01 and *p < 0.05).

By 48 h post-EHV-1 inoculation, IL-8 expression was statistically significantly upregulated in all Flu Avert/EHV-1-inoculated ERECs when compared with untreated/mock-inoculated ERECs for all Flu Avert treatment timepoints (Figures 5A–D). IL-8 was also upregulated in untreated/EHV-1-inoculated ERECs for all treatment timepoints, and this was statistically significant for treatment days 1, 5, and 7. For CCL2 expression, a similar trend was observed in ERECs 48 h post-EHV-1 inoculation that were pre-treated with Flu Avert on days 2, 5, and 7, but this trend was not statistically significant (Figures 5F–H). No appreciable trend was observed in ERECs that were pre-treated with Flu Avert on day 1 (Figure 5E). The expression of CCL5, CXCL9, and CXCL10 was not different between groups 48 h post-EHV-1 infection (data not shown).

**Figure 6:**
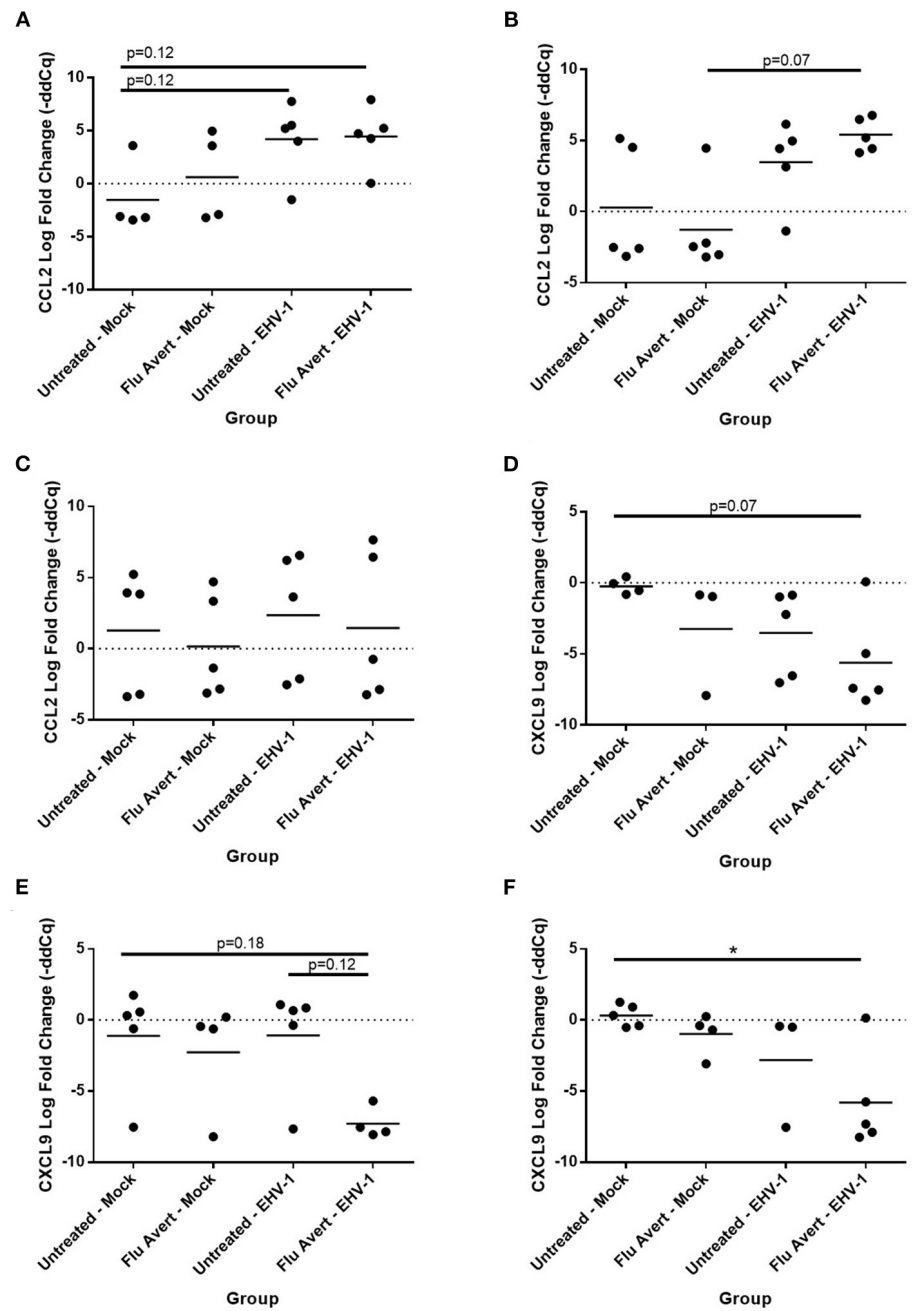
Effect of Flu Avert treatment on chemokine mRNA expression in ERECs collected from five horses 72 h following EHV-1 inoculation. **(A)** CCL2 mRNA expression in ERECs that were treated with Flu Avert on day 2 prior to EHV-1 inoculation. **(B)** CCL2 mRNA expression in ERECs that were treated with Flu Avert on day 5 prior to EHV-1 inoculation. **(C)** CCL2 mRNA expression in ERECs that were treated with Flu Avert on day 7 prior to EHV-1 inoculation. **(D)** CXCL9 mRNA expression in ERECs that were treated with Flu Avert on day 2 prior to EHV-1 inoculation. **(E)** CXCL9 mRNA expression in ERECs that were treated with Flu Avert on day 5 prior to EHV-1 inoculation. **(F)** CXCL9 mRNA expression in ERECs that were treated with Flu Avert on day 7 prior to EHV-1 inoculation. The mean log fold change (–ddCq) is represented by a bar (*p < 0.05).

By 72 h post-EHV-1 infection, IL-8 was upregulated in EHV-1-inoculated ERECs pretreated with Flu Avert that was statistically significant for treatment days 2 and 5 and untreated/EHV-1-inoculated ERECs for all treatment days besides for day 1 (data not shown). A similar trend (although not statistically significant) was observed for CCL2 expression where EHV-1 inoculation increased CCL2 compared with untreated/mock-inoculated cells in cells pre-treated with Flu Avert on day 2 (*p* = 0.12; Figure 6A) and day 5 (*p* = 0.07; Figure 6B), but this increase for CCL2 was not observed in ERECs pre-treated with Flu Avert on day 7 (Figure 6C). In addition, ERECs treated with Flu Avert and inoculated with EHV-1 showed downregulated expression of CXCL9, and this was statistically significant in cells treated with Flu Avert 7 days prior to inoculation with EHV-1 (day 2 *p* = 0.07, Figure 6D; day 5 *p* = 0.18, Figure 6E; day 7 *p* < 0.05, Figure 6F). Furthermore, there was a downregulation in CXCL9 in EHV-1-inoculated ERECs treated with Flu Avert 5 days prior compared with untreated EHV-1-infected cells (*p* = 0.12; Figure 6E). There were no differences in mRNA expression of CCL2 or CXCL9 between ERECs for Flu Avert treatment day 1 (data not shown). There were no differences in the expression of CCL5 and CXCL10 between groups at 72 h post-EHV-1 inoculation (data not shown).

Expression for IFNβ was low or near the limit of detection for all samples, and only ~17% of all samples were positive for this cytokine. Based on the low percent of positive samples, no relative quantitation was performed. However, EHV-1-infected samples appeared to be more likely to express IFNβ than mock-inoculated samples. Of the 40 samples with detectable IFNβ mRNA expression, 30 were EHV-1-inoculated wells. The expression of IFNα, IFNγ, and IL-10 mRNA was positive in <10% of the total samples (data not shown).

**Figure 7:**
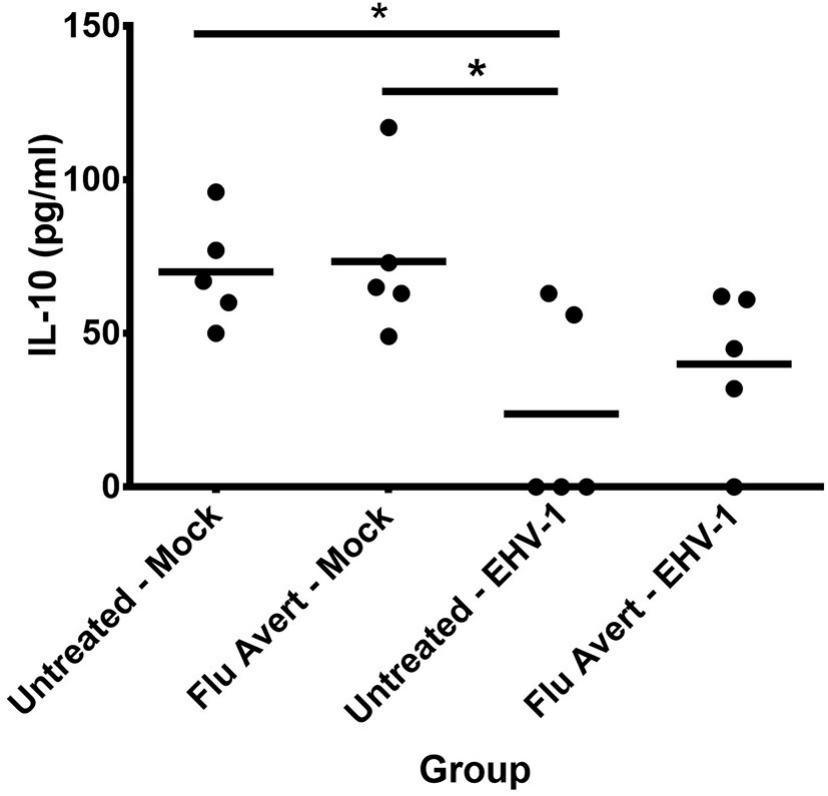
IL-10 protein expression in EREC supernatants collected from five horses. ERECs were treated with Flu Avert (or left untreated) on day 7 prior to EHV-1 inoculation, and supernatants of cultures were collected 72 h post-EHV-1 infection. The mean concentration (pg/ml) is represented by a bar (*p < 0.05).

Finally, EHV-1 inoculation downregulated IL-10 protein expression in EREC supernatants 72 h post-EHV-1 inoculation in both Flu Avert and untreated cells when compared with mock-inoculated ERECs for treatment days 1, 2, and 5, but this was not statistically significant (data not shown). Interestingly, for treatment day 7, there was less downregulation of IL-10 observed in the supernatants from cells pre-treated with Flu Avert prior to EHV-1 inoculation when compared with the mock-inoculated groups (Figure 7). There were no differences in IL-17 protein expression between groups (data not shown). The expression of IFNα and IL-4 proteins was below the limit of detection of the assay, and the expression of IFNγ proteins was below 4 U/ml in all samples (data not shown).

## Discussion

Our hypothesis was that treatment of ERECs to a LAIV would stimulate innate immunity, and that this response would also protect cells against EHV-1 infection and replication. We found that Flu Avert stimulated epithelial immunity, and that these responses were optimal starting at day 5 post-Flu Avert treatment. While it has been well-established in humans that influenza virus infection or LAIV vaccination stimulates mucosal immunity, very little work has been done evaluating the equine respiratory epithelial innate immune response to EIV or equine LAIV. Here, we show that Flu Avert treatment of ERECs stimulated the induction of chemokine expression. In particular, we observed an upregulation of IL-8 and CCL2 expression following Flu Avert treatment, which agrees with other studies investigating mucosal chemokine responses to influenza vaccination. In humans, it has been shown that following LAIV treatment, chemokine expression for CXCL9, CXCL10, CCL5, CCL2, and IL-8 is upregulated in human primary epithelial cell cultures (45, 46).

Our data, along with these human studies, indicate that LAIVs, such as Flu Avert, act to stimulate epithelial mucosal immunity in primary cells of the upper respiratory tract and may provide important information for how the natural airway will respond to treatment in the horse. In contrast to the human cell culture studies, we did not observe any effect on CCL5 or CXCL10 mRNA expression in our equine system. This may be attributed to different responses of human and equine cells to LAIV treatment, or be due to limits of detection in our system that was used at 37°C (the optimal temperature for EHV-1 replication *in vitro*), whereas the human studies were conducted at 32°C, which is known to more closely mimic the temperature of the human upper respiratory tract (60). It is known that Flu Avert is a cold-adapted vaccine, and previous work indicates that Flu Avert replicates more efficiently at 30°C in Madin-Darby Bovine Kidney (MDBK) cells (61). Because the magnitude of innate immune responses in epithelial cells depends on the replication of viral RNA, optimal replication of LAIV may be necessary for optimal induction of chemokines (45), but since we depended on viable cell cultures for the subsequent EHV-1 inoculation, a suboptimal temperature for Flu Avert replication of 37°C was chosen. However, even at suboptimal temperatures for Flu Avert replication, we did observe induction of IL-8 and CCL2. In addition, CXCL9 was downregulated in this experiment. Clearly, more work is needed to test Flu Avert treatment in horses and its effect on chemokine responses in the *in vivo* nasal mucosa and further downstream immune responses.

Stimulation of innate immunity is classically considered to occur quickly, often within hours, following virus infection. However, our data suggest that following treatment with Flu Avert, peak responses start at 5 days post-treatment. This could be due to slow replication in the ERECs due to attenuation of the Flu Avert virus. In a study of LAIV treatment in human nasal epithelial cell cultures, it was also observed that peak cytokine responses occurred several days following treatment (45). It has been shown that peak cytokine responses to LAIV correspond to the expression of viral RNA in human epithelial cultures and *in vivo* nasal IFNα peaks correspond with peak EIV viral titer and clinical disease (46, 62). While we did not measure Flu Avert RNA genome titers over time, it is likely that these peaked in ERECs at a similar timepoint where peak cytokine expression was observed.

In addition to chemokines, EIV infection is known to stimulate interferon production in nasal secretions of ponies beginning 2 days post-infection (43). In our study, interferon expression was near the detection limit, and we only detected interferon mRNA (IFNβ) expression in ERECs following treatment with Flu Avert in a few samples. In human epithelial cells, it is shown that LAIV stimulates many features of the interferon pathway, including the upregulation of pattern recognition receptors and expression of interferon-stimulated genes (ISGs) (45, 46). However, in a cohort of human patients who received LAIV, only 21% had detectable levels of IFNα in nasal wash, whereas several ISGs were upregulated (47). These results, along with ours, indicate that while the interferon pathway is stimulated by LAIV, the temporal regulation of different genes likely contributes to the timing of expression and ultimately detection of mRNA or proteins. The interferon pathway is stimulated through activation of pattern recognition receptors, including TLR3 (19, 44), and in ERECs, we have previously shown that either infection with wild-type EIV or treatment with Flu Avert stimulated the expression of TLR3 within 24 h (61). In our study, it is likely that the interferon pathway was stimulated in ERECs in response to Flu Avert; however, IFNα and IFNβ as well as IFNγ were at or below the limit of detection in our cultures. Though it has been shown that LAIV induces many aspects of the interferon pathway in epithelial cells, there is evidence that this response is more attributed to the induction of type III interferon (IFNλ), which is unique to epithelial cells (63), rather than type I (IFNα, IFNβ) interferons. Similarly, in mice, it was found that IFNλ was induced in epithelial cells to far greater levels than type I interferons in response to influenza challenge (64). Future studies should consider analyzing the expression of IFNλ or ISGs in addition to type I and II interferons in order to get a more complete picture of the role of Flu Avert on the interferon response.

Interestingly, peak chemokine modulation that occurred in ERECs on 5–8 days post-Flu Avert treatment also corresponded with optimal reduction in EHV-1 titers in the EHV-1-inoculated cells. The delayed peak in cytokine expression explains the timing of optimal EHV-1 reduction in our system, which would be the ultimate goal of Flu Avert treatment in horses. Here, the effects of Flu Avert on EHV-1 replication were investigated by generating EHV-1 intracellular and extracellular growth curves in ERECs. Our data suggest that treating with Flu Avert 5 days prior to EHV-1 inoculation was most likely to interfere with EHV-1 replication. However, the reduction in copy number was not complete, and there was a large amount of variability, which is likely because the EREC system lacks the comprehensive immune system that the whole respiratory tract possesses. While antiviral cytokines and chemokines are expressed in ERECs, the leukocyte recruitment that would occur in the natural airway is absent, and this recruitment and activation of leukocytes is critical for effective innate and adaptive immune responses and for effective pathogen elimination. Despite this limitation, the EREC system was useful at providing information about the epithelial response to viral infection and to determine an idea of optimal timing for follow-up studies in the natural host aimed at evaluating viral titers, protection, and induction of innate and adaptive immunity.

The intention of this work was to investigate the suitability of the equine mucosal influenza virus vaccine to stimulate strong non-specific innate antiviral respiratory immunity to provide protection from another major equine respiratory virus, EHV-1. This could be particularly helpful because it is known that EHV-1 modulates and suppresses innate respiratory immunity (20, 22, 33–39). In this study, it was observed that Flu Avert treatment modulated chemokine expression in EHV-1-infected cells. These chemokine responses of epithelial cells to EHV-1 are instrumental in promoting immune cell recruitment to the upper respiratory epithelium and ultimately protection from EHV-1 (14, 16, 65). Specifically, we observed that ERECs treated with Flu Avert showed a significant upregulation of IL-8 expression by 24 h post-EHV-1 inoculation, whereas this increase was not seen in the untreated cells until 48 h post-EHV-1 inoculation. Influenza virus infection is known to stimulate IL-8 production in epithelial cells, supporting our finding that pre-treatment with Flu Avert enhanced IL-8 responses to EHV-1 in our study (48). Similar to IL-8 responses, we observed that Flu Avert treatment prior to EHV-1 inoculation enhanced the CCL2 expression in response to EHV-1. CCL2 and CCL5 act to recruit monocytes and memory T-cells, and influenza virus infection is known to stimulate both CCL2 and CCL5 production in epithelial cells (66, 67). Our data suggest that pre-treatment with Flu Avert may act similarly and might function to rescue CCL2 expression in respiratory epithelial cells during EHV-1 infection (66). The modulation of IL-8 and CCL2 by EHV-1 is supported by studies showing that EHV-1 proteins, gG, and pUL56 suppress IL-8 protein responses and neutrophil chemotaxis and play an important role in viral clearance (22, 38, 39). Furthermore, EHV-1 pUL56 has been shown to suppress the expression of CCL2 in ERECs and in peripheral blood mononuclear cells (PBMCs) following EHV-1 infection (22, 24). The current theory is that EHV-1 selectively modulates the recruitment of immune cells to its advantage by suppressing IL-8 and CCL2 and preventing the recruitment of cells for viral clearance but not interfering with the recruitment of cells that permit an establishment of viremia (36). Interestingly, it has also been shown that neuro-pathogenic EHV-1 strains recruit monocytes *via* CCL2 and CCL5 expression more significantly than less neuro-pathogenic strains (28).

CXCL9 and CXCL10 are related chemokines that serve to recruit T-cells, including CTLs, and are thus of interest when considering the induction of a robust immune response to herpesviruses (68). In our study, we found that CXCL10 expression was enhanced in Flu Avert-treated, but not media-treated, cells 24 h following EHV-1 inoculation. On the other hand, we were surprised to observe a downregulation of CXCL9 in Flu Avert-treated cells following EHV-1 inoculation. A previous study in ERECs showed a significant increase in both CXCL9 and CXCL10 expression following EHV-1 inoculation (27). It has been shown that dramatic CXCL9 and CXCL10 expression is induced in PBMCs following EHV-1 infection, but this expression peaked at 10 h post-inoculation and returned to baseline by 24 h post-inoculation (24). Furthermore, influenza virus has been shown to induce rapid the expression of CXCL9 and CXCL10 following infection in epithelial cells (69). It is unclear why we observed an upregulation of CXCL10, but a downregulation of CXCL9 in ERECs following Flu Avert treatment and EHV-1 inoculation, given the related function of these two chemokines. One explanation is that our study investigated responses several days following initial Flu Avert treatment and not until 24 h post-EHV-1 infection; thus, it is possible that the timepoints we investigated here were too late to observe an upregulation of CXCL9 expression. More work is needed to fully understand the complicated balance of these chemokine expression patterns following epithelial infection with Flu Avert and EHV-1 and its implications for EHV-1-related disease. However, it is clear that timing post-Flu Avert administration must be considered in order to see the optimal response.

Previous works in ERECs have shown an increase in type I interferons (IFNα and IFNβ) in response to EHV-1 infection (20, 22, 26). In our study, levels of IFNα mRNA and proteins were below detectable levels. One explanation is that the previous studies were performed using EHV-1 at an MOI of 10. In the present study, the inoculations were performed at a MOI of 1, which is more biologically relevant. Additionally, interferons are well-known to be quickly and transiently expressed molecules of the innate immune system. In one study, Poelaert et al. found that type I interferon proteins were detectable as early as 10 h post-EHV-1 inoculation in ERECs and remained detectable up through 72 h (26). In our study, we measured mRNA, and thus it is possible that the peak mRNA expression period in our system was missed with the timepoints chosen for sample collections. Additionally, type III, rather than type I, interferons are known to be specialized for epithelial responses and have been shown to be the primary drivers of the interferon responses to influenza virus infection (63, 64). To our knowledge, IFNλ expression has not been evaluated in equine respiratory tissues and may play a role in protection from EHV-1 infection.

Finally, IL-10 is an immunoregulatory cytokine that tempers excessive immune responses and is important for preventing immune-mediated damage to host tissues (70). In our study, IL-10 mRNA was below the limit of detection of our assay; however, we were able to detect IL-10 proteins in EREC supernatants following EHV-1 infection. We found that EHV-1 infection downregulated IL-10 expression; nevertheless, this response was less apparent in those cells treated with Flu Avert 7 days prior to EHV-1 inoculation, indicating that Flu Avert treatment at this time may have counteracted the suppressive effect of EHV-1. In addition to its anti-inflammatory role, IL-10 is known to contribute to B-cell activation, which at the site of the nasal epithelium could contribute to mucosal IgA antibody production (71). Our finding that Flu Avert may reduce the IL-10 suppression by EHV-1 may be important when considering boosting mucosal humoral immunity.

In summary, this study revealed important information regarding the epithelial innate immune response to LAIV in ERECs. In this primary cell culture system, peak responses to this attenuated virus occurred several days following administration, and this correlated with reduction in EHV-1 replication. These results suggest that Flu Avert may be effective at counteracting the immune-modulatory properties of EHV-1; however, more work is needed to understand whether the chemokine enhancing effects of Flu Avert at the site of the epithelium will translate to more protective adaptive immune responses *in vivo*. Finally, our study suggests that future *in vivo* research should consider the timing of administration of immune stimulants or mucosal vaccines during experimental design.

## Data Availability Statement

The raw data supporting the conclusions of this article will be made available by the authors, without undue reservation.

## Ethics Statement

The animal study was reviewed and approved by Institutional Animal Care and Use Committee of Michigan State University.

## Author Contributions

LZ and GS contributed to the study execution, data analysis, and preparation of the manuscript. All authors contributed to the study design, interpretation of data, and approval of the final manuscript.

## Conflict of Interest

WV, DB, and FB are employed by MSD Animal Health. The remaining authors declare that the research was conducted in the absence of any commercial or financial relationships that could be construed as a potential conflict of interest.
